# Insights from the predicted structural analysis of carborane substituted withaferin A with Indoleamine - 2,3-dioxygenase as a potent inhibitor

**DOI:** 10.6026/97320630012374

**Published:** 2016-11-30

**Authors:** Syed Hussain Basha, Abhishek Thakur, Firoz A Samad

**Affiliations:** 1 Innovative Informatica Technologies, Hyderabad, Telangana – 500 049, INDIA; 2 Birla Institute of Technology Mesra, Ranchi- 835215, INDIA; 3 Department of Chemistry, University of Miami, Coral Gables, Florida 33124, USA; 4 Center for Genetics and Inherited Diseases, Taibah University, PO Box 3001, Medina, Saudi Arabia

**Keywords:** Carboranes, Withaferin-A, Indoleamine-2, 3-dioxygenase, Anti-cancer, immunotherapy, molecular modeling, HOMO, LUMO, docking, molecular dynamic simulations

## Abstract

Indoleamine-2,3-dioxygenase (IDO) an immunoregulatory enzyme and emerging as a new therapeutic drug target for the treatment of cancer. Carboranes, an icosahedral arrangement of eleven boron atoms plus one carbon atom with unique pharmacological properties such low toxicity, isosterism with phenyl ring and stability to hydrolysis. On the other hand, carboranes are known to increase the interaction of ligand with non-polar region of the protein provides an excellent platform to explore these carboranes towards designing and development of novel, potent and target specific drug candidates with further enhanced binding affinities. Despite of their many potential applications, molecular modeling studies of carborane-substituted ligands with macromolecules have been rarely reported. Previously, we have demonstrated the promising high binding affinity of Withaferin-A (WA) for IDO. In this present study, we investigated the effect of carborane substitutions on WA compound towards developing novel analogs for target specific IDO inhibition with better potency. Interesting docked poses and molecular interactions for the carborane substituted WA ligands were elucidated. Based on our In-silico studies, carborane substituted at various position of WA has shown enhanced binding affinity towards IDO, worth of considering for further studies.

## Background

Recently boron based drug design has gained an interest in medicinal chemistry, especially carboranes. Its unique pharmacological properties, low toxicity, iso-sterism with phenyl ring, stability to hydrolysis, low toxicity provides an excellent platform to explore towards novel drug discovery along with alteration of existing drug scaffolds. Carboranes are an icosahedral arrangement of eleven boron atoms plus one carbon atom, improving the stability and metabolism of drug [1]. The unique icosahedral and rigid cluster of carboranes makes it chemically most stable cluster of atoms in all of chemistry. Moreover the large surface area of carborane increases the interaction of ligand with non-polar region of the protein [2]. Carboranes do not cure any disease directly, however they greatly enhance the binding capability of drug more tightly to its target and are an attractive surrogate of cyclohexyl or lipophilic phenyl ring in novel drug designing approaches [3].

Indoleamine-2,3-dioxygenase (IDO) one of the most important immunoregulator enzyme responsible for metabolism of tryptophan as part of Kynurenin pathway. Tryptophan is catabolized in the tumor tissue by the rate-limiting enzyme IDO expressed in tumor cells or antigen presenting cells [4]. First carborane based drugs targeting IDO states that carboranes cage can be well tolerated by IDO enzyme in cell free assay and suggested that larger lipophilic drugs can be explored towards getting potent IDO inhibitor [5]. Withaferin-A (WA) an active constituent of Withania somnifera has shown to possess a wide range of therapeutic index for various cancer physiological states. In our recent study, we have demonstrated that Withaferin-A is a potential IDO inhibitor [6]. In this present study, we have designed ten novel carborane substituted Withaferin A compounds, and demonstrated their enhanced target specific IDO inhibition capability.

## Methodology

### Softwares and programs

The ligands containing carborane cluster were generated by MarvinSketch v5.12.3 [7] and were saved to pdb format for further processing. All ligands were Energy minimized by Avogadro v1.1.1 [8] using “Auto optimization tool” by applying ‘UFF’ force field with steepest descent algorithm. The popular docking program AUTODOCK v4.0 [9] was chosen for molecular docking of the carborane substituted ligands. Argus lab 4.0.1 [10] was used to carry out HOMO and LUMO orbital analysis of the compound Withaferin A. Desmond v3.6 Package [11] was used for molecular dynamic simulation studies as per the default protocol [12,13], with 425,000 Å3 orthorhombic periodic boundary conditions buffered at 10 Å distances with predefined TIP3P water model [14] using OPLS2005 force field [15]. System was minimized and relaxed using default protocol before running the 25 ns NPT production simulation [16].

### Molecular modeling and preparation strategy of the carborane substituted ligands

In case that one or two hydrogens (“H”) of carborane are flipped into the cavity of the cluster in the MarvinSketch, the hydrogens were deleted and re-added to generate proper icosahedral carborane structures. In order to stabilize the carborane substituted ligands a small time step of about 100ps of “Molecular Dynamics” was applied followed by Energy minimization. Thus obtained energy-minimized structures were saved as the pdb file and were transferred to Autodock for docking studies. The PDB files generated by Marvin Sketch, however, were only partially readable by Autodock. Certain bonds were missing in the carborane clusters displaying only fivefold coordinated carbon and boron atoms but the overall geometries of the carborane structures were conserved. Missing bonds were added using Autodock and reconstructed cluster geometries were saved as ‘pdbqt’ files.

### Molecular docking strategies

Initially docking was not successful in Autodock program due to the non-availability of required force field parameterization of the boron atom, which is not provided by this software packages in default settings. This is a major drawback in applying computational chemistry as a predictive tool for synthetic chemists in studying molecular interactions of carborane containing compounds with macromolecules in the process of designing novel drug compounds. However, this hurdle can be crossed by applying a simple but effective modification of converting all boron atoms ‘B’ in ligands to carbon atoms ‘C.3’ by editing the AD4.1_bound.dat file and rest of the docking protocol was of default as explained elsewhere [17]. Preprocessing of protein and ligand structure for docking calculations as per the default protocols followed elsewhere [18]. Each autodock calculation was run thrice to check the convergence of the results.

### HOMO and LUMO orbitals

Geometry optimized Withaferin A compound was selected under the “Single point Energy” calculation in order to carry out Hamiltonian Quantum mechanics PM3 calculations by selecting the surface properties as HOMO and LUMO. The grid box was centered at -4.04, -8.75 and -5.68 and the size of the grid box was set to 40 Å XYZ respectively.

## Results & Discussion

### HOMO and LUMO orbitals guided designing of carborane substituted Withaferin A derivatives

**Figure 1:**
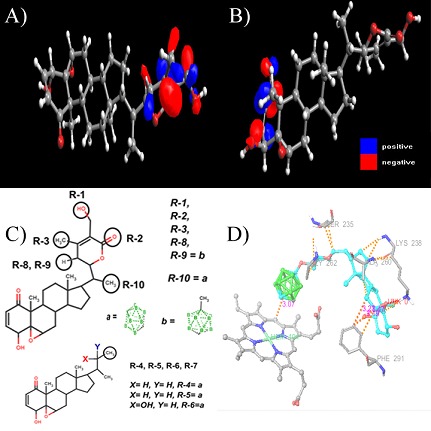
a) HOMO and b) LUMO orbitals of Withaferin A c) designing of derivative Withaferin A compounds with carborane substituted at various positions. d) Docking snapshot of compound 01 showing interactions with heme moiety along with some key residues at the active site of the IDO.

Chemical reactivity of a compound can be revealed theoretically using the HOMO (Highest Occupied Molecular Orbital) and LUMO (Lowest Unoccupied Molecular Orbital) orbitals, which are commonly known as Frontier Orbitals. Electrophilic and nucleophilic attacks were shown to correlate very well with atomic sites having high density of the HOMO and LUMO orbitals respectively. We have conducted HOMO and LUMO calculations in order to determine the most favorable sites in the Withaferin A for carborane substitution. HOMO and LUMO orbitals of Withaferin A compound are shown in Figure 1a , b. The positive and negative phases of the orbital are represented by the two colors, the blue regions represent an increase in electron density and the red regions a decrease in electron density. HOMO site of Withaferin A compound was found to be more suitable for carborane substitution compared to LUMO, moreover, HOMO site is the part of the compound which was found to be most interacting with the active Heme moiety of the IDO as revealed in our previous study [6]. Based on this information, we have designed 10 derivative Withaferin A compounds with carborane substituted at various positions as depicted in figure 1c.

### Molecular docking

The results of docking simulations demonstrated strong binding affinity and promising IC50 values with good interactions for critical residues present in IDO’s catalytic site with binding energies between -8.64 and -13.62 kcal/mol (Table 1). Molecular interactions for each of the docking snapshot has been charted in Table 2 and shown in Figure 3. As per the docking simulations, compound 04 has shown the least binding affinity of -8.64, whereas compound 01 has shown the best binding affinity of -13.62 kcal/mol with a pIC50 of 843.78 picomolar (Figure 1d). The present studied compounds, especially compound 01 with carborane ring substituted at R1 position has shown far better binding energies compared to the native withaferin A compound -11.51 kcal/mol with 3.63 nanomolar of pIC50 as revealed in our earlier study [6]. On the other hand, compound 03 and compound 10 has shown to be next best inhibitory potential with -11.63 and -11.55 Kcal/mol binding energy and pIC50 value of 3.0 and 3.43 nano molar respectively. As per these results, all the novel carborane substituted withaferin A compound derivatives are quite promising IDO inhibitors, especially compound 01. Moreover, these results are also highly in support of the claims that carborane increases the binding affinity for the compounds. When the docking snapshot of best binder compound 01 in complex with IDO was analyzed, it was found to be forming 11 direct hydrogen bonds with Gly265, Gln266, Gly261, Gly236, Arg297, Tyr298 which are way stronger than the three hydrogen bonds formed with SER167; LYS377 and HEME moiety for the Withaferin A compound without any carborane substitution. Apart from direct hydrogen bonds, compound 01 was also found to be forming hydrophobic bond with Tyr298; polar and charged bonds with Gln266 and Arg297 residues respectively. All the molecular interactions for each of the compound have been charted out in Table 2.

### MD simulations of IDO in complex with compound 01

Previously we have studied the IDO protein dynamics in water solvent in its native state without any ligand along with in presence of native withaferin A compound [6]. In this present study, we have taken the best carborane substituted withaferin A derivative compound 01 in complex with IDO with the binding energy of −13.62 kcal/mol obtained using autodock for further validation using MD simulations with the aim of revealing the influence of carborane substitution on withaferin A compound to bind IDO. Statistically significant results of the MD simulations are tabulated in Table 3. Each simulation was run twice to check for the convergence.

**Figure 2:**
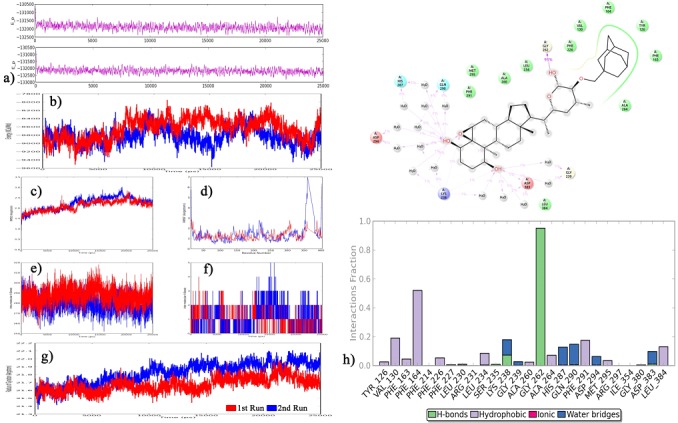
a-g) Simulation event analysis graphs of the IDO in complex with compound 01 h) Molecular interactions network of the compound 01 in complex with IDO during the 25ns simulation time scale.

**Figure 3:**
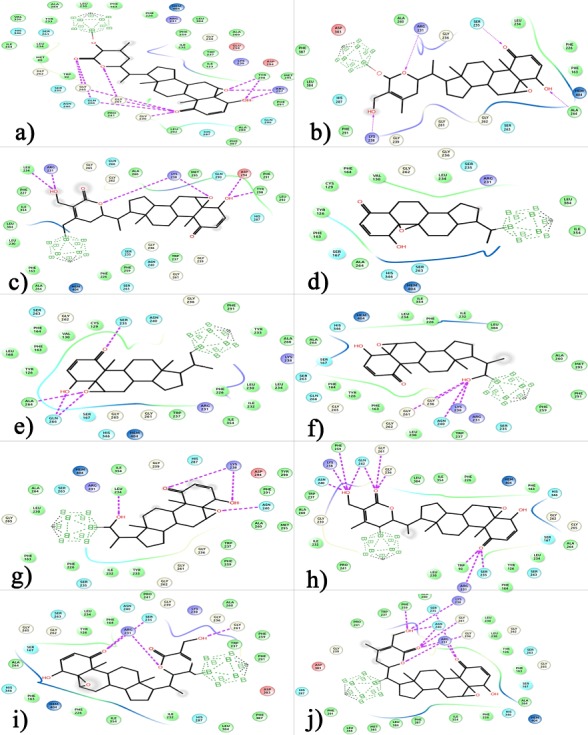
Molecular interactions of designed compounds 1-10 (a-j) docked inside the binding pocket of IDO.

As part of the analysis, we have analyzed the total potential energy of the simulated system containing protein in complex with compound 01 and it was observed to be maintaining an average of −131500 and −132000 kcal/mol of energy (Figure 2a), which is well minimized in comparison to IDO’s averaged energy of −10,739 and −10,834 kcal/mol in presence of no ligand in both first and second simulation, respectively as revealed during our previous study [6]. On the other hand, protein’s total energy in presence of compound 01 was found to be maintaining an average of -9000 kcal/mol of energy in both cases, however was found to upto -9600 kcal/mol in case of 2nd run (Figure 2b). The results in Figure 2c show that the RMSDs of the trajectories for the complex were well below 3.0 Å comparable with IDO in its apo state (Table 3).

When IDO’s residue fluctuations were calculated in presence of compound 01, it was observed that the majority of the highly active residue movements in its apo state have been minimized in presence of compound 01 compared to native WA. However, during the second run of the simulation, a sudden opening of the binding cavity has been observed due to the positional flip of residues between 350-360 in IDO, allowing sliding of the compound deep inside the active binding site, facilitating more interactions for stronger binding affinity as evident with Figure 2d. We also calculated the total number of intra molecular hydrogen bonds present within the IDO in complex with compound 01 throughout the simulation time accounting for its stability and found out that it is maintaining an average of 279 and 287 during the first and second run respectively (Figure 2e). Whereas, when the intermolecular hydrogen bonds between IDO and compound 01 during first and second run of the simulation was analyzed; 0-5 and 0-4 range was observed respectively with a mean of atleast 1 hydrogen bond holding throughout the simulation (Figure 2f). ROG graph (Figure 2g) of the complex has evidenced that the protein IDO has slightly expanded as the simulation progresses during the second simulation run comparatively by maintaining an average of 21.8 and 21.6 Å (Table 3). During the simulation, a total of 26 contacts were present between compound 01 and IDO, frequent direct H-bonds were observed with residues GLY262, LYS238 and ASP383 with upto 95% occupancy during MD trajectory. Apart from direct hydrogen bonds, compound 01 was also found to be forming various hydrophobic contacts with residues TYR126, VAL130, PHE163, PHE164, PHE214, PHE226, PHE227, ARG231, LEU234, ALA260, ALA264, PHE291, MET295, GLY380 and LEU384 with no ionic bonds but few residues LEU230, ARG231, LYS238, GLY239, HIS287, GLN290, ASP294, ARG297, ILE354 and ASP383 which are found to be forming water bridges as shown in Figure 2h. These results are highly in support to the strong inhibiting and stabilizing potential of compound 01 compared to native WA on IDO.

## Conclusion

The present study provides a rationalization to the ability of carborane substitution to enhance the binding capability of the native Withaferin A compound. Our computational analysis evidenced that the large negative values of binding energy is involved in binding of novel designed carborane substituted WA derivatives with the IDO consolidating this complex’s thermodynamic stability; moreover, predicted IC50 values further substantiated our hypothesis that these compounds has the potential to inhibit IDO. Several interesting molecular interactions with residues at the active binding site of IDO were revealed to be the major factor for these complex formations. Carborane was found to be increasing molecular interactions several folds contributing to the overall binding capabilities of the present tested compounds. The present theoretical evaluations are a step further towards enhancing our knowledge on how these carborane substitutions at various positions on a given compound can enhance the potential of the compound binding capabilities to the specific drug target of interest.

## Authors’ contribution

Conceived and designed the experiments: SHB and AT; Performed the experiments: SHB and AT; analysis of the results: SHB, AT and FAS; Drafting and editing of the manuscript: SHB, AT and FAS. All the authors have read and approved the manuscript.

## Figures and Tables

**Table 1:** Docking results of carborane substituted Withaferin A compound derivatives with IDO.

S. No	Ligand name	1st Run		2nd run		3rd run	
Binding energy	Inhibition constant	Binding energy	Inhibition constant	Binding energy	Inhibition constant
1	1	-13.12	947.25 pM	-13.62	843.78 pM	-13.56	892.61 pM
2	2	-10.55	18.51nM	-10	47.10nM	-10.74	13.40nM
3	3	-11.63	3.0nM	-11.6	3.16nM	-11.2	6.14nM
4	4	-8.67	439.96nM	-8.69	424.42nM	-8.64	466.35nM
5	5	-10.77	12.67nM	-10.32	27.5nM	-10.78	12.45nM
6	6	-9.43	122.79nM	-9.41	126.76nM	-9.43	122.23nM
7	7	-9.46	117.31nM	-9.5	108.18nM	-9.46	116.65nM
8	8	-10.35	25.81nM	-10.32	27.35nM	-10.34	26.17nM
9	9	-10.6	17.0nM	-11.62	3.04nM	-10.1	39.42nM
10	10	-11.55	3.43nM	-11.55	3.43nM	-11.54	3.50nM

**Table 2:** Molecular interaction of carborane substituted Withaferin A compound derivatives with IDO

Derivative no	No of H bonds	H-bonds forming residues	Hydrophobic (Green)	Polar (Sky blue)	Charged (-ve) Red	Charged (+ve) Purple	Glycine (Yellow)
1	11	Gly265, Gln266, Gly261, Gly236, Arg297, Tyr298	Tyr298	Gln266	--	Arg297	Gly265, Gly261, Gly236
2	4	Lys238, Arg231, Ser235, Ala264	Ala264	Ser235	--	Lys238, Arg231	--
3	6	Leu234, Arg231, Lys238, Asp294, Tyr298	Leu234, Tyr298	--	Asp294,	Arg231, Lys238	--
4	--	--	--	--	--	--	--
5	4	Ser234, Gln266, Ala264	Ala264	Ser234, Gln266	--	--	--
6	4	Gly261, Gly236, Lys238, Asn240	--	Asn240	--	Lys238	Gly261, Gly236
7	4	Leu234, Lys238, Asn240	Leu234	Asn240	--	Lys238	
8	10	Phe259, Lys238, Asn240, Gln242, Gly261, Gly236, Trp92, Arg231, Ser235	Phe259, Trp92	Asn240, Gln242, Ser235	--	Lys238, Arg231	Gly261, Gly236
9	4	Arg231, Ser235, Gly261	--	Ser235	--	Arg231	Gly261
10	12	Phe259, Ser235, Lys238, Asn240, Arg231, Gly236, Gly261	Phe259	Ser235, Asn240	--	Lys238, Arg231	Gly236, Gly261

**Table 3:** MD simulation statistics for the compound 01 in complex with IDO

MD run	Total energy (Kcal/mol)		Intra H-Bond		Inter H-Bonds		RMSD		ROG	
Range	Mean	Range	Mean	Range	Mean	Range	Mean	Range	Mean
Run 1	-9574 to -8136	-8832	251 to 307	279	0 to 5	1	0.0 to 3.0	2.2	21.3 to 22.1	21.8
Run 2	-9516 to -7948	-8643	255 to 318	287	0 to 4	1	0.0 to 2.8	2.1	21.4 to 21.9	21.6
